# Current and alternative trends in antibacterial agents used in mammalian semen technology

**DOI:** 10.21451/1984-3143-AR2019-0111

**Published:** 2020-03-17

**Authors:** Caio Sérgio Santos, Alexandre Rodrigues Silva

**Affiliations:** 1 Departamento de Ciências Animais, Universidade Federal Rural do Semi-Árido, Mossoró, RN, Brasil

**Keywords:** antibiotic, bacteria, chilling, cryopreservation, extenders

## Abstract

The use of antibacterial substances as additives in extenders for ensuring the sanitary quality of the semen employed in reproductive biotechniques and preserving it from bacterial deterioration has been reported since the mid-twentieth century. However, the deleterious effects of these drugs on the sperm quality as well as their effectiveness in controlling bacterial growth in the preserved semen have been questioned. The aim of this review was to report the antimicrobials primarily used in the extenders added to the semen of mammals, and to present alternatives to their use. Among the various mammalian species, there is a large variation regarding the antimicrobial types added to semen extenders as cephalosporins (ceftiofur, cefdinir, eg) and quinolones (ofloxacin, ciprofloxacin), alone or in combination with large action spectra substances as penicillin-streptomycin and gentamicin-tylosin-lincomycin-spectinomycin. To combat problems related to bacterial resistance to these drugs, the emergence of alternatives is increasingly evident. Among these alternatives, use of physical methods as centrifugation and filtration, as well as the use of antimicrobial peptides and other substances from different origins have been highlighted for presenting antimicrobial potential.

## Introduction

Reproduction plays an important role in ensuring the efficiency of animal production (Woelders et al., 2012), maintaining their biodiversity, and supporting the conservation programs of vulnerable or threatened species (Costa and Martins, 2008). Thus, assisted reproductive techniques such as artificial insemination (AI) associated with semen technology allow the preserved male genetic material to be used in females that are isolated from the males. This maximizes the availability of the germplasm and facilitates genetic improvement, disease control and conducting of reproductive procedures regardless of time and geographical location (Morrell and Mayer, 2017).

Although one of the benefits of AI is to reduce the spread of diseases, this technique may allow the pathogens to be easily disseminated through the transmission of contaminated semen (Foote, 2002). The semen can be contaminated particularly during the process of its collection or cryopreservation if good practices are not adopted (Zampieri et al., 2013). Moreover, the presence of pathogens can damage the sperms (Diemer et al., 2000), adversely affecting the quality of the semen subjected to cryopreservation.

For these reasons, in order to prevent bacterial multiplication, antibacterial substances are usually added to the diluents during the cryopreservation procedures. Contrarily, some of these substances can affect the survival of sperm, the use of which can sometimes be restricted due to active regulations. Moreover, their indiscriminate use can lead to bacterial resistance, even when utilized in small quantities (Morrell and Wallgren, 2011). At this sense, this review aims to report the main antimicrobial agents added to mammalian semen extenders and the factors related to their effectiveness, as well as presenting alternatives to their use.

## Sources and consequences of bacterial contamination in semen technology

Some microorganisms are present in the semen because they cross the bloodstream of the animals suffering from bacteremia. Others may come from the preputial microbiota of healthy semen donors and associate themselves with the semen during ejaculation and collection. Contamination can also occur during the processing and storage of semen, which may be derived not only from the environment but also from substances (particularly of animal origin, such as egg yolk) added to the semen extenders, equipment and materials such as storage bottles, which are in direct or indirect contact with the semen (Thibier and Guerin, 2000). Liquid nitrogen is an effective cryopreservant of pathogens and may also be an unsuspected source of contamination. This emphasizes on the need for care during the storage of semen, which should be carried out using well-protected and sealed straws (Mazurova and Krpatova, 1990).

Microorganisms can cause serious economic damages by reducing the semen quality and possible dissemination of pathogens (Prieto-Martínez et al., 2014). Contaminated semen can reduce the conception rate, cause early embryonic death and/or endometritis, clinical diseases in herds, and/or infections by unwanted pathogens (Maes et al., 2008).

It has been reported that microbes affect and interfere with the semen quality. It was verified that the presence of bacteria can cause morphological alterations, acrosome exocytosis (Prieto-Martínez et al., 2014), sperm agglutination, decreased sperm motility and membrane integrity (Sepúlveda et al., 2014), decrease in the sperm longevity within 48 h of collection and processing (regardless of whether the diluent used is for short, medium or long term), and also causes acidification of the medium (pH between 5.7 and 6.4) (Althouse et al., 2000).

In general, it is known that the semen collection process is far from being a sterile procedure because of the involvement of multiple sources that can lead to bacterial contamination (Bussalleu and Torner, 2013). In this sense, additional measures such as regular monitoring of the animals and semen, biosafety measures to reduce contamination during collection, processing and storage, and the treatment of semen with the appropriate antimicrobials (Maes et al., 2008) are necessary.

## International regulations

Several countries require the assessment of animal health for the importation of semen obtained from production animals like cattle, buffaloes, goats, sheep, horses and pigs. The regulations recommend the use of antimicrobials in semen extenders as a measure to prevent the spread of diseases and sperm degradation.

With the development of the semen trade using farm animals, regulations set by the European Union and the European Council (EEC), which required the use of antibacterial cocktails at inseminating doses, emerged in the late 1980s and early 1990s. According to the Directives 88/407 and 90/429 of the European Council, Annex C2, which lays down the health policy requirements for intra-community trade and imports of bovine and porcine semen, respectively, it is stated that an effective combination of antimicrobials, particularly against leptospires and mycoplasmas, should be added to the semen after the final dilution (European Union, 1988, 1990). The EEC directive 92/65 (European Union, 1992), which applies to other animals such as Equidae, swine and small ruminants, reports another combination of antimicrobials consisting of gentamicin (250 μg/mL), tylosin (50 μg/mL) and lincomycin/spectinomycin (150/300 μg/mL), or amikacin (75 μg/mL) and divecacin (25 μg/mL), which can be used alone (EEC directive 92/65).

Some Latin American countries also have a legislation establishing the minimum sanitary requirements for the processing and marketing of animal semen. For instance, in Brazil, laws were established only more than a decade after the implementation of the European regulations. Normative instructions for cattle (Brasil, 2003), goats and sheep (Brasil, 2014) recommended the same combination of substances used by the European Directives. However, regulations concerning the importation of swine and equine semen from the Mercosur countries neither define the substances nor the doses that may be added to the diluents.

### Use of antibacterial substances in semen extenders

The antimicrobials frequently used in mammalian semen diluents include the β-lactams (penicillins, cephalosporins), which interfere with the process of bacterial cell wall synthesis, causing lysis and cell death (Spinosa et al., 2011). Others include the aminoglycosides (gentamicin, streptomycin, amikacin), macrolides (tylosin, spectinomycin) and lincosamides (lincomycin), which are inhibitors of bacterial protein synthesis (Spinosa et al., 2011). Among the various mammalian species, however, there is a large variation regarding the antimicrobial types and concentrations added to extenders used for both chilling or freezing semen procedures.

Studies on bacterial control in ruminants (Table 1) date from the 40s and 50s in the United States of America, emphasizing the importance of its use in the development of artificial insemination (AI) in bovine. Initially, the antimicrobials most used in bovine semen technology were penicillin and streptomycin (Almquist et al., 1949). Almquist (1951) reported their use at concentrations of 1000 IU/mL and 1000 μg/mL, respectively, in an egg yolk citrate diluent and their combined use demonstrated better herd fertility results than when used alone. Two years later, Alford (1953) evidenced that diphtheroid bacilli demonstrated resistance even when high concentrations of streptomycin were added to the bovine semen. Nowadays, the use of a combination of gentamicin-tylosin-lincomycin-spectinomycin (500 μg/mL-100 μg/mL–300 μg/mL–600 μg/mL, respectively) is largely recommended for diluting the semen of several species, including bulls and buffaloes (Andrabi et al., 2001; Akhter et al., 2008; Andrabi et al., 2016). In spite of this, other antimicrobial combinations, as the ceftiofur/tylosin (200 μg/mL-100 μg/mL) and ofloxacin (100 μg/mL) have been highlighted for a more effective control of the bacterial growth in bovine semen (Gloria et al., 2014). Besides, the use of ciprofloxacin (600 μg/ml) added to the Tris-citric acid diluent has been also recommended for the preservation of buffaloes’ semen (Akhter et al., 2013). On the other hand, the use of cephalosporin as cefdinir (1 mg/mL) and cefoperazone sodium (1 mg/mL) was effective for controlling bacteria during ram semen chilling at 5 °C up to 96 h (Azawi and Ismaeel, 2012). In the same species, the use of streptomycin-penicillin combination was more effective to bacterial control than lincomycin and sulfadiazine, without causing negative effects on sperm (Moustacas et al., 2010).

**Table 1:** Some antimicrobial substances successfully used in mammalian semen technology.

	Antimicrobials	Concentrations	Semen diluents	Storage temperatures	Storage times	References
Bulls	Streptomycin and penicillin	100 to 1000 μg/mL or IU/mL of each	Sodium citrate-egg yolk	4,5°C	20 days	Almquist et al. (1949)
	Streptomycin and penicillin (alone or combination)	1,000 mg/mL and 1,000 IU/mL	Sodium citrate-egg yolk	-	-	Almquist (1951)
	Gentamicin, tylosin, lincomycin and spectinomycin	500 μg/mL, 100 μg/mL, 300 μg/mL and 600 μg/mL	Tris-citric acid	-196 °C	1 day	Andrabi et al. (2001)
	Ceftiofur and tylosin	200 μg/mL and 100 μg/mL	Bioxcell CSS I and II	-145 °C	7 days	Gloria et al. (2014)
	Ofloxacin	100 μg/mL	Bioxcell CSS I and II	-145 °C	7 days	Gloria et al. (2014)
Buffaloes	Gentamicin, tylosin, lincomycin and spectinomycin	500 μg/mL, 100 μg/mL, 300 μg/mL and 600 μg/mL	Skimmed milk based	5 °C	3 days	Akhter et al. (2008)
	Streptomycin and penicillin G	1,000 mg/mL and 1,000 IU/mL	Skimmed milk based	5 °C	3 days	Akhter et al. (2008)
	Ciprofloxacin	600 μg/mL	Tris-citric acid	-196 °C	-	Akhter et al. (2013)
	Gentamicin, tylosin, lincomycin and spectinomycin	500 μg/mL, 100 μg/mL, 300 μg/mL and 600 μg/mL	Tris-citric acid	-196 °C	1 day	Andrabi et al. (2016)
Rams	Streptomycin and penicillin G	1,000 mg/mL and 1,000 IU/mL	Tris-glucose	-196 °C	-	Moustacas et al. (2010)
	Gentamicin	250 µg/mL	Tris-glucose	-196 °C	-	Moustacas et al. (2010)
	Cefdinir	1.0 mg/mL	Sodium citrate-fructose-egg yolk	5 °C	4 days	Azawi and Ismaeel (2012)
	Cefoperazone sodium	1.0 mg/mL	Sodium citrate-fructose-egg yolk	5 °C	4 days	Azawi and Ismaeel (2012)
Boars	Gentamicin	-	-	18°C	3 days	Mazurová and Vinter (1991)
	Gentamicin and florfenicol	100 μg/mL and 100 μg/mL	Biosolwens Plus	15 °C	10 days	Bryła and Trzcinska (2015)
	Gentamicin	250 µg/mL	Beltsvile Thowing Solution	17 °C	3 days	Waberski et al. (2019)
	Gentamicin	250 µg/mL	Androstar Premium	17 °C	3 days	Waberski et al. (2019)
	-	-	Androstar Premium ^*^	5 °C	3 days	Waberski et al. (2019)
Stallions	Amikacin	2,500 µg/mL	Tris-egg yolk	4 °C	7 days	Arriola and Foote (1982)
	Amikacin or Ticarcillin	2,000 µg/mL	Skim milk-glucose	23 °C and 5 °C, respectively	1h and 2 days, respectively	Jasko et al. (1993)
	Gentamicin or polimixin B	100 µg/mL or 1,000 IU/mL, respectively	Skim-milk glucose	20 °C and 5 °C, respectively	8h and 2 days, respectively	Vaillancourt et al. (1993)
	Penicillin G and amikacin or Ticarcillin-clavulanic acid or Ceftiofur	1,000 IU/mL and 1,000 µg/mL or 1,000 µg/mL or 1,000 µg/mL	Skim-milk glucose	5 °C	1 day	Varner et al. (1998)
	Gentamicin	250 µg/mL	EquiPro^©^	15 °C	4 days	Price et al. (2008)
	-	-	EquiPro^©^ *	5 °C	4 days	Price et al. (2008)
	Cefquinome	0.99 mg/mL	EquiPro^©^	5 °C	2 days	Parlevliet et al. (2011)
	Ticarcillin-clavulanic acid	0.5, 1.0 and 1.5 mg/mL	INRA 96^®^ **	5 °C	3 days	Olivieri et al. (2011)
	Penicillin G and amikacin	As supplied by the manufacturer	VDMZ	5 °C	3 days	Olivieri et al. (2011)
	Penicillin G and gentamicin	1,000 IU/mL and 1,000 mg/mL	BotuSemen^®^	5 and 15 °C	1 day	Ramires Neto et al. (2015)
	Penicillin G and amikacin	1,000 IU/mL and 1,000 mg/mL	TAMU	Fresh and 5 °C	1 day	Hernández-Avilés et al. (2018)
	Meropenem	1,000 mg/mL	TAMU	Fresh and 5 °C	1 day	Hernández-Avilés et al. (2018)
	Penicillin G and amikacin	1,000 IU/mL and 1,000 mg/mL	INRA 96^®^ ^**^	Fresh and 5 °C	1 day	Hernández-Avilés et al. (2019)
Dogs	Gentamicin, tylosin, lincomycin and spectinomycin	250, 50, 150 and 300 μg/ml; 500, 100, 300 and 600 μg/ml; and 1000, 200, 600 and 1200 μg/ml	Tris-citric acid-fructose-egg yolk	5 °C	3 days	Becher et al. (2013)
Koala (*Phascolarctos cinereus*)	Penicillin G and gentamicin	1,000 IU/mL and 100 µg/mL	PBS	16 °C	1 day	Johnston et al. (1998)
Collared peccaries (*Pecari tajacu*)	Streptomycin and penicillin G	1,000 mg/mL and 1,000 IU/mL	Tris-citric acid-fructose-egg yolk	5 °C	1,5 days	Santos et al. (2019b)
	Gentamicin	70 µg/mL	Tris-citric acid-fructose-egg yolk	5 °C	1,5 days	Santos et al. (2019a)

* Antibiotic-free diluents;

** It contains penicillin (105 μg), streptomycin (38 μg) and amphotericin B (0.315 μg) on its composition.

As early as 1968, studies had indicated that the bacteria isolated from porcine semen were also resistant to some antibacterials such as penicillin-streptomycin (Almond and Poolperm, 1996). At this sense, in the 1990s, Mazurová and Vinter (1991) reported a decrease in bacterial contamination (<10^3^) of boar semen after dilution in a gentamicin-treated diluent incubated at 18 ° C for up to 72h. This study was pioneering at comparing gentamicin with several other antimicrobials such as ampicillin, apramycin and cefoxitin. Currently, gentamicin has established itself as one of the most used antimicrobials in boar semen diluents (Schulze et al., 2017). However, Gączarzewicz et al. (2016) demonstrated that the inhibitory activity of even gentamicin may be limited during long-term preservation (16°C for five days) in diluent (X-cell^®^). In fact, the storage time is an important factor related to the amount of antimicrobial in the boar semen extender. Thus, there are short-term (1 to 3 days) (Johnson et al., 1982) and long-term (more than 4 days) extenders (Haugan et al., 2007) and antibacterial concentrations are generally higher in these latter types (Table 1).

In general, the types of antimicrobials addressed in studies on stallion semen conservation are the most varied (Table 1). Arriola and Foote (1982) highlighted that bacterial strains present in equine ejaculates were resistant to common antimicrobials such as penicillin and streptomycin. Moreover, it was demonstrated that polymyxin B (Jasko et al., 1993) and gentamicin (Aurich and Spergser, 2007) could negatively affect motility parameters in cooled stallion spermatozoa, despite being potent antimicrobials in semen extenders (Vaillancourt et al., 1993). However, this information was recently contradicted by Price et al. (2008) that reported that the addition of small amounts of gentamicin (250 μg/mL) reduced bacterial growth and improved the sperm motility, velocity and viability in the stallion semen stored at 15 °C up to 96 h, compared to that of the control, which did not contain any antibacterial substance. Anyway, the cefquinome (0.99 mg/mL), a fourth-generation cephalosporin, was demonstrated as a suitable substitute for gentamicin within 48 h preservation of equine semen at 5 ºC, providing both the bacterial control and the maintaining of sperm parameters (Parlevliet et al., 2011). Recently, Ramires Neto et al. (2015) reported that the penicillin-gentamicin combination (1,000 IU-1,000 mg/mL) in BotuSemen^®^ (BS) (a skim milk based diluent) yielded lower bacterial load in stallion semen after cooling, compared with INRA 96^®^, a commercial diluent, which already contains penicillin (105 μg), streptomycin (38 μg) and amphotericin B (0.315 μg) in its composition. In fact, it was reported that the addition of clavulanic acid-associated ticarcillin (Timentin^®^) would be more effective for the bacterial control during equine semen preservation than the isolate use of INRA 96^®^ diluent (Olivieri et al.,2011). In addition, the use of the potassium penicillin-amikacin combination has also been evidenced for providing efficient antibacterial action and maintaining sperm parameters during equine semen storage (Varner et al., 1998; Hernández-Avilés et al., 2018; Hernández-Avilés et al., 2019).

For companion animals, protocols for the use of antimicrobials in semen technology are generally extrapolated from other domestic animals and studies focused on the determination of effective antimicrobial concentrations are rare. For instance, Barbosa et al. (2010) found that the isolate use of penicillin at 500 IU, 1000 IU and 1500 IU / mL concentrations during canine semen cryopreservation did not control bacterial growth after thawing. Due to the worries on the *Mycoplasma* sp. and *Ureaplasma* sp dissemination through canine semen exchange, Becher et al. (2013) addressed the comparison of the effect of two antibiotic combinations, as benzylpenicillin (0.6 g/L) plus streptomycin (1.0 g/L) and the gentamicin-tylosin-lincomycin-spectinomycin (GTLS) combination at increasing concentrations (GTLS-1: 250, 50, 150 and 300 μg / ml; GTLS-2: 500, 100, 300 and 600 μg / ml; GTLS-3: 1000, 200, 600 and 1200 μg / ml), which was demonstrated for being more effective regarding the microbial control.

Studies describing the use of antimicrobials in the preservation of semen from wild animals (Table 1) are even more scarce than in companion animals. The combination of penicillin (1000 IU/mL) and gentamicin (100 μg/mL) was effective at preserving koala semen (*Phascolarctos cinereus*) at 16 °C for 24 h as it prevented bacterial growth without interfering on the sperm motility (Johnston et al., 1998). Additionally, the addition of gentamicin (70 μg / mL) (Santos et al., 2019a) and a combination of penicillin (2000 IU/mL and 1000 IU/mL)-streptomycin (2 mg/mL and 1 mg/mL) (Santos et al., 2019b) to the semen of collared peccaries (*Pecari tajacu*) allowed the control of bacterial growth in the samples and did not present toxic effects on the quality of the chilled semen maintained up to 36 h.

Due to the differential action of antimicrobial substances in the distinct species, various studies have been conducted in order to stablish appropriate antimicrobial concentrations in the diluent. The experimental design of the studies does not follow a standardized pattern, and includes several variables, such as type and concentration of antimicrobials, preservation time, storage temperatures, previous inoculation with pathogenic bacteria and fertility trials. In this context, it is evident that the temperature is an important factor that can interfere on bacterial dissemination during semen storage, particularly for stallion and boar cooled-semen technology, in which protocols highlight use of a relatively high temperature (15 to 17 °C) in a nutrient-rich extender that can favor bacterial growth. However, it was demonstrated that even for stallions (Price et al., 2008) and boars (Waberski et al., 2019), the hypothermic storage (5 °C) may reduce the use of antimicrobial drugs (Table 1).

### Alternatives to the use of antibacterial agents

The development of bacterial resistance against the main antibacterial agents used in semen extenders, such as the combination of penicillin and streptomycin (Sone et al., 1982), amoxicillin, gentamicin, lincomycin, tylosin and spectinomycin (Althouse and Lu, 2005), has been reported. Thus, the search for alternatives that overcome bacterial resistance is a reality. Alternatives include antimicrobial peptides, physical methods for reducing bacterial load and the use of various substances, whether animal, plant or other origins.

#### Antimicrobial peptides

Recently, research has been focused on the use of antimicrobial peptides (AMP) that may destabilize the bacterial cell wall (Table 2). Bussalleu et al. (2017) have investigated the use of the proline-arginine-rich antimicrobial peptide, PR-39, which belongs to the group of porcine myeloid antimicrobial peptides 36 (PMAP-36) and 37 (PMAP-37), as an additive to porcine semen extenders. The authors observed that PMAP-37 at 0.5, 1 and 3 μM concentrations reduced the bacterial load up to 10 days, besides improving the sperm viability. Moreover, the PR-39 (20 μM) promoted bacterial inhibition but it was found to be cytotoxic to the porcine sperm, whereas PMAP-36 did not exhibit any antimicrobial action.

**Table 2:** Alternative methods for bacterial control in semen samples from mammalian species.

Alternatives	Types	Animals	References
Antimicrobial peptides	PR-39 (proline-arginine-rich antimicrobial peptide)	Boar	Bussalleu et al. (2017)
	PBD-1 (beta defensin-1) and PBD-2 (beta defensin-2)	Boar	Puig-Timonet et al. (2018)
	ε-Polylysine	Boar	Shaoyong et al. (2019a)
Physical methods	Single-layer centrifugation (SLC)	Boar	Morrell and Wallgren (2011), Morrell et al. (2019)
	Single-layer centrifugation (SLC)	Stallion	Morrell et al. (2014), Guimarães et al. (2015)
	Microfiltration	Boar	Barone et al. (2016)
Miscellaneous substances	Royal jelly	Bull	Abd-Allah (2010)
	*Aloe vera* gel	Ram Collared peccary (*Pecari tajacu*) Bull	Brito et al. (2014) Souza et al. (2016) Farias et al. (2019)
	Kojic acid	Boar	Shaoyong et al. (2019b)
	Iodine methionine	Boar	Fang et al. (2017)
	BactiBag^®^	Boar	Camugli et al. (2019)
	Sodium alginate	Buffalo	Kumar et al. (2019)

Other peptides of porcine origin such as beta defensin-1 (PBD-1) and beta-defensin-2 (PBD-2) may be used as antimicrobial agents. Puig-Timonet et al. (2018) found that both peptides 3 mM concentration did not impair the viability and motility of the spermatozoa and were able to control microbial growth to some extent. Similarly, ε-Polylysine (40 to 128 mg/mL) was shown for effectively inhibiting bacterial growth, improving sperm quality and *in vitro* fertilization, being able to replace 50% of the gentamicin used in the extender (Shaoyong et al., 2019a).

#### Physical methods

Some physical methods as centrifugation and filtration (Table 2), which may reduce or replace antimicrobial use, have also been highlighted. An alternative method reported by Morrell and Wallgren (2011) was the use of single-layer centrifugation using Androcoll™-P, a colloid based on glycidoxypropyltrimethoxysilane-coated silic, that completely removed the bacteria from 60% of the samples of swine semen and reduced the bacterial load in 40%.

Recently, the possibility of separating all sperm from seminal plasma without affecting the semen quality was investigated in swine (Morrell et al., 2019). The ejaculates were diluted in the antimicrobial-free Beltsville Thawing Solution and subjected to single-layer centrifugation in a low-density colloid, which provided an increase on sperm velocity and linearity, besides removing or reducing bacterial contamination in boar ejaculates.

In equine, Morrell et al. (2014) also evaluated the removal of bacteria by single-layer centrifugation using Androcoll™-E after the addition of bacteria (*E. coli*, *Klebsiella pneumoniae*, *Streptococcus equi* subsp. *zooepidemicus*, *Taylorella equigenitalis*, among others) in different proportions in the aliquots of semen. The reduction in counts ranged from 68% to 97% among bacteria. In another experiment involving equine semen, it was also found that colloidal centrifugation using Androcoll™-E before freezing reduced the total bacterial load after thawing and positively influenced the post-thaw motility (Guimarães et al., 2015).

Another physical method recently described for antimicrobial reduction is the seminal plasma (SP) microfiltration. The SP was separated of the sperm by centrifugation, then filtered with a 1.2 µm syringe prefilter (NalgeneTM) followed by a 0.22 µm syringe filter (NalgeneTM), and added in 20% of swine fertilization medium with and without antibiotic for AI doses. This process, in addition to reduce bacterial contamination in boar semen, has improved some parameters such as motility, plasma membrane and acrosome integrity, and mitochondrial activity (Barone et al., 2016).

#### Other alternative substances

Some recent studies have been considering the use of new natural bioactive products (Table 2) such as phytotherapics and other compounds obtained from plants or animals to replace antibiotics in semen technology.

Abd-Allah (2010) found that the use of 0.4% royal jelly in the cryopreservation of bovine semen improved the viability and fertility characteristics of the spermatozoa. Although the study did not focus on the use of an antimicrobial activity of the jelly, it has a known antimicrobial component namely 10-hydroxy-2-decenoic acid (Blum et al., 1959), which may also have influenced the results and requires to be investigated.

Another interesting alternative would be the use of *Aloe vera*, which presents among its constituents a non-volatile fraction with bactericidal action (Radha and Laxmipriya, 2014). Its use was recently reported as an efficient cryoprotectant for ovine (Brito et al., 2014), collared peccary (Souza et al., 2016) and bovine semen (Farias et al., 2019), but its antimicrobial potential during semen preservation was not yet investigated. Besides *Aloe vera*, the *Ocimum gratissimum* leaf extract at 0.5% (Alaba and Sokunbi, 2018) as well as the essential oils of *Malaleuca alternifoli*a and *Rosmarinus officinalis,* both at 0.4 mg/mL concentration (Elmi et al., 2019), have shown satisfactory antimicrobial potential in the preservation of wild boar semen.

Other alternative to the antimicrobial drugs includes substances of various origins (Table 2) as the Kojic acid (5-hydroxy-2-hydroxymethyl-1,4-pyrone), which is a weakly acidic secondary metabolite produced by aerobic fermentation of *Aspergillus* and *Acetobacter* fungi (Song et al., 2019). It was demonstrated for inhibiting bacterial growth (at concentrations of 20 to 100 mg/mL) in diluted swine semen and for improving (40 mg/mL) sperm quality, sperm capacitation, number of sperm attached to oocyte and embryonic development (Shaoyong et al., 2019b).

Additionally, the iodine methionine, a new type of chelate amino acid, was demonstrated for inhibiting the proliferation of the phylum Proteobacteria and the genus *Staphylococcus* as well as *Pseudomonas*, and for improving sperm motility, plasma membrane integrity and acrosome integrity in swine semen after 6 days storage (Fang et al., 2017). Recently, the IMV laboratories reported the use of the BactiBag^®^, a semen bag with bacteriostatic molecules, which shown potential for control bacterial growth during porcine semen storage for 3 days (Camugli et al., 2019).

Finally, the addition of sodium alginate to egg-yolk diluent improved the metal chelating capacity and antibacterial properties of the extender, besides improving antioxidant and cryoprotective activities during the cryopreservation of buffaloes semen (Kumar et al., 2019).

## Final considerations

The problem of bacterial resistance has been reported since the mid-twentieth century and has stimulated the development of several studies that seek to test new antibacterial agents. However, to date, there are few well-designed studies that have aimed to evaluate the bacteriostatic/bactericidal characteristics of the antimicrobials, their effects on the semen quality of animals, and the time of bioactivity of these compounds under conditions of chilling and freezing.

In general, a variety of antibacterial substances have shown satisfactory results for ruminants and equines, in which antimicrobial drugs are used alone or in combination to increase the spectrum of action. On the other hand, the addition of gentamicin to the porcine semen diluent is well established, but in small and wild animals, studies are scarce and constitute a prominent area for further experimentation.

In parallel, the search for antimicrobial alternatives has been increasing; however, further research is needed to enable the use of adequate concentrations of these new compounds which should be effective for bacterial without impairing the sperm quality. Furthermore, in the long term, the *in vivo* effect of these substances on fertility after the use of chilled or cryopreserved semen by AI should be evaluated.
